# When DNA Mutations Interplay with Cellular Proliferation: A Narrative History of Theories of Carcinogenesis

**DOI:** 10.3390/cancers16112104

**Published:** 2024-05-31

**Authors:** Laura El Nachef, Audrey Bouchet, Michel Bourguignon, Nicolas Foray

**Affiliations:** 1 Inserm U1296 Unit, “Radiation: Defense, Health and Environment”, 28 Rue Laennec, 69008 Lyon, France; laura.el-nachef@inserm.fr (L.E.N.); audrey.bouchet@inserm.fr (A.B.); michel.bourguignon@inserm.fr (M.B.); 2 Département de Biophysique et Médecine Nucléaire, Université Paris Saclay, Versailles St. Quentin-en-Yvelines, 78035 Versailles, France

**Keywords:** cancer, carcinogenesis, tumor, history of sciences

## Abstract

**Simple Summary:**

The current theories of carcinogenesis are the result of a long succession of thoughts and beliefs since antiquity. From the humor theory that interpreted cancer as an excess of black bile to the three notions of initiation, promotion, and progression and the most recent definitions of hallmarks of cancer, we reviewed and discussed each of these steps to better understand the state of the art of carcinogenesis.

**Abstract:**

While cancer is one of the most documented diseases, how normal cells become cancerous is still debated. To address this question, in the first part of this review, we investigated the long succession of theories of carcinogenesis since antiquity. Initiated by Hippocrates, Aristotle, and Galen, the humoral theory interpreted cancer as an excess of acid, the black bile. The discovery of the circulation of blood by Harvey in 1628 destroyed the basis of the humoral theory but revived the spontaneous generation hypothesis which was also promoted by Aristotle. In 1859, the theory of microbes promoted by Pasteur demonstrated the irrelevance of this last theory and contributed to the emergence of the germ cancer theory, opposed to the cellular theory of cancer, in which cancer was supposed to be caused by microbes or transformed cells, respectively. These theories were progressively refined by the notions of initiation, promotion, and progression thanks to advances in mutagenesis and cellular proliferation. In the second part of this review, recent discoveries and paradigms in carcinogenesis, notably the role of the protein ATM, a major actor of the stress response involved in both mutagenesis and cellular proliferation, were discussed to better understand the current state of the art of carcinogenesis.

## 1. Introduction

**Figure 1:**
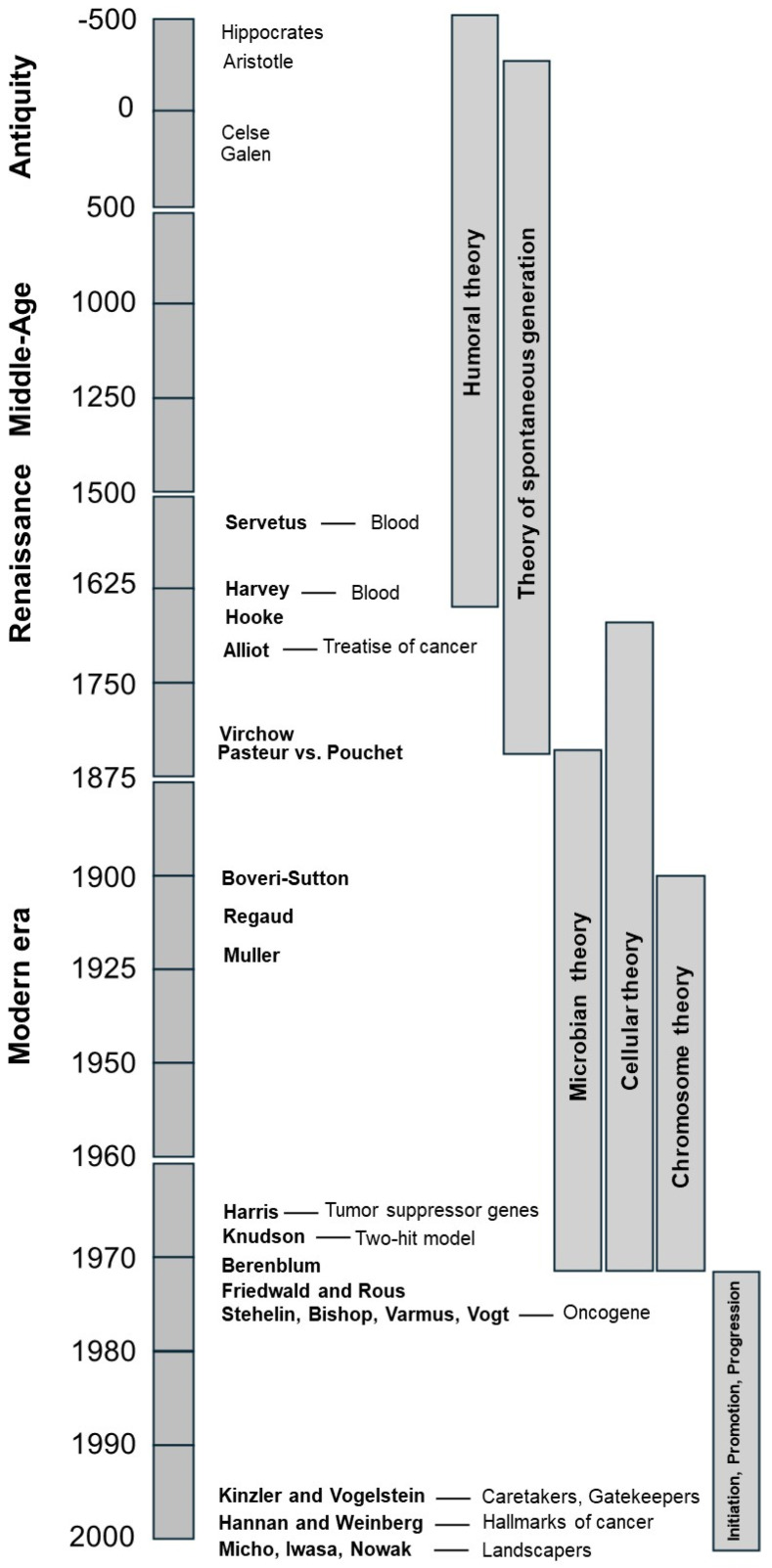
Chronology of ideas evolution about carcinogenesis.

To date, cancer is one of most frequent and documented diseases in the world [1]. However, the explanation of how normal cells become cancerous has been debated since antiquity [2,3,4,5,6]. The current theories of carcinogenesis are the results of a long succession of thoughts, beliefs, and technological developments during more than two thousand years. Here, we proposed to review the different steps and models that have led from the humoral theory to the triptychs “initiation, promotion and progression” of the somatic mutation theory of carcinogenesis by focusing on the evolution of ideas about cancer formation throughout history. In the first part of this review (Section 2, Section 3, Section 4, Section 5, Section 6 and Section 7), the different models of carcinogenesis proposed from antiquity until the 2000s were reviewed through an approach that was deliberately intended to be epistemological and not an exhaustive catalog of clinical and biological descriptions of tumor types or treatments. The aim of this part was to better understand how scientific achievements have led to successive paradigms about cancer (Figure 1).

In the second part of this review (Section 8 and Section 9), the recent discoveries and paradigms related to carcinogenesis including our model based on the radiation-induced nucleoshuttling of the protein ATM, a major actor of the individual stress response involved in both mutagenesis and control of cellular proliferation, were discussed to better understand the current state of the art of carcinogenesis.

## 2. From Antiquity to 1850, Cancer Was Explained by the Humoral Theory

### 2.1. The Cancer Origin in Antiquity: The Legacy of Hippocrates

**Figure 2:**
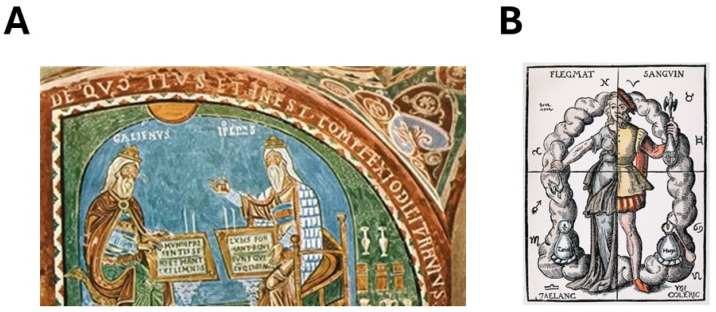
The humoral theory. (**A**) Fresco of the beginning of 13th century representing Hippocrates and Galen, crypt of the Santa-Maria Cathedral of Anagni, Italy. (**B**) Painting from Quinta Essentia by Leonhard Thumeysser (1574) representing the humoral theory.

According to the Greek mythology, a crab was sent by Hera, sister and wife of Zeus, to distract the attention of Heracles during his fight against the nine-headed Lernaean Hydra, the second of his twelve labors. Heracles crushed the crab with his heel. In the 5th century BC, possibly inspired by this episode and the mental picture of the crab (*cancer* in Greek), Hippocrates (Figure 2) emphasized the analogy between the crab and a breast tumor by describing it as a central spot (crab head) with a net of vascular twigs (crab legs) [7]. He considered that any disease, including cancer, was the result of a change of healthy habits (*cacoethes*) and the “direct environment”. Since the term “cacoethes” means “bad state” in ancient Greek, and by interpreting further the term “direct environment”, one can attribute to Hippocrates the hypothesis that cancer may be due to an imbalance caused by “endogenous” or “exogenous“ factors [7]. Although many ancient physicians (including Egyptians, Mesopotamians, Greeks, and Romans) described different types of tumors and treatments, very few, if any, have provided a basic explanation of their origin, leaving the field open to divine will or predestination to explain the intrinsic origins of such imbalance.

In his *De Medicina* (about medicine), written in the 1st century AC, Aulus Cornelius Celsus strengthened the observations of Hippocrates and supported the paradigm that cancer is a succession of ordered clinical steps with, at the beginning, the *cacoethes*, interpreted as a kind of pre-tumor (or predisposition?), that can progress from non-ulcerous to an ulcerous carcinoma to finally provide a verrucous lesion, the *thymion* [8]. Like Hippocrates, Celsus insisted on the fact that the cancer at its first step (*cacoethes*) is operable while it is not in the next steps of progression [8]. It is noteworthy that the notion of malignancy (benign or malignant tumor) was not introduced yet.

### 2.2. The Triumph of Humoral Theory: The Definitions of Galen

Some years after Celsus, Claudius Galenus (Galen) (Figure 2), in his *Epitomas*, provided a significant breakthrough in the descriptions, the definitions and the understanding of numerous clinical features concerning cancer and tumors [9]. In his work *De tumoribus praeter naturam* (“the tumors against nature”), Galen introduced a clear definition of the tumor in the first sentences of the book [9]:

*“A tumor is an increase in length, width and depth occurring in a part of the body. There is obviously no question of calling the natural distension of fat and plump crops that way; what characterizes the unnatural tumor is that it thwarts the action of the part of the body that it affects.”*

By developing the first ideas of Hippocrates, Galen popularized the humoral theory that was based on the hypothesis that the body contains precise and balanced proportions of blood, phlegm, yellow bile, and black bile (Figure 2). Each humor corresponds to the four human temperaments: sanguine, phlegmatic, choleric, and melancholic, respectively. Health is defined as the state in which each of these humors is well balanced and in correct proportions (the *crasis* state). By contrast, pathologies originate from humor excesses or deficiencies (the *dyscrasis* state) [9]. Particularly, in *De tumoribus praeter naturam*, Galen wrote the following:

*“If the bile spreads into the flesh, if it is acrid, it eats away the surrounding tissue and ulcerates it. If it is without acridity, it produces cancer without ulceration.”*

Hence, both Hippocrates and Galen supported the idea that the dyscrasis at the origin of the formation of the tumor may be due to *extrinsic factors* such as the seasons change, water, air, and winds, and by intrinsic factors such as age, physical conditions, and race [9] (Figure 2).

### 2.3. From the Dark Ages to the Age of Enlightenment: The Breast Cancer of Ann of Austria

Such explanations remain unchanged until the 17th century, while a plethora of treatments, mainly based on bloodletting, were continuously proposed from antiquity to the Age of Enlightenment. During this period, the texts of Galen were considered as the indisputable reference in medicine (like those of Aristotle in physics): the ban on analyzing corpses limited investigations and progress in medicine.

**Figure 3:**
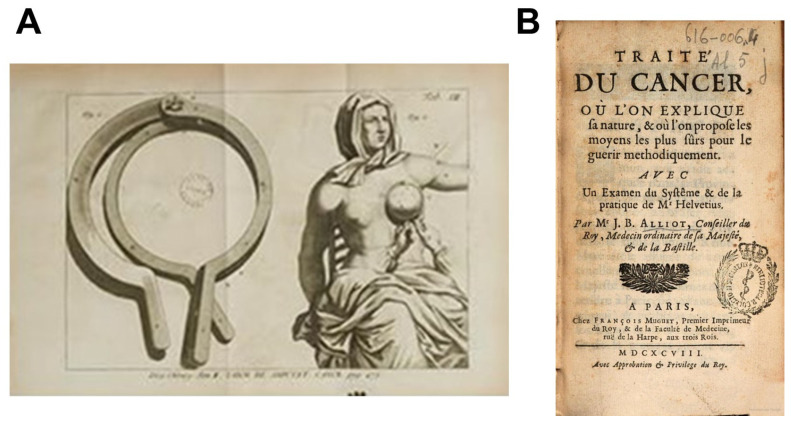
The perception of cancer in the 17th century. (**A**) Drawings representing the system of slices that permits to cut the dead flesh after a treatment of breast cancer (from *De cancro mammarum*, from Jacques Dupuy, 1768). (**B**) The first page of the Traité du Cancer from Jean-Baptiste Alliot, edited under the protection of the Sun King Louis XIV (1698) [10].

A historical event upset this established order: the breast cancer of Anne of Austria, queen of France, widow of King Louis XIII and mother of the Sun King Louis XIV. In November 1664, Anne of Austria suffered from cancer in her left breast. Her first physician ordered some bloodlettings to relieve pain but humbly declared that he was unable to cure the queen. Louis XIV therefore undertook an incredible research campaign on treatments for breast cancers, and doctors, priests, or charlatans paraded to propose treatments of all kinds. Among them, the physicians Pierre Alliot and his son, Jean-Baptiste proposed in April 1665 a therapy based on an ointment made of arsenic. This ointment was supposed to kill tumor tissue, and a system of slices was developed to remove the dead flesh thereafter (Figure 3). Although both corrosive and painful, such treatment produced the only rest of the queen before her death in January 1666 [10].

### 2.4. The First Treatise on Cancer

Jean-Baptiste Alliot benefited from the transitional prestige of his father and was appointed physician of the king. The King Louis XIV ordered him to write a treatise on cancer which can be considered as a general summary of all the current knowledge about cancer and tumors of that time (Figure 3) [10]. In this book, published in 1698, Jean-Baptiste Alliot responded to the expectations of the scientific community which was no longer satisfied with the theories of Hippocrates, Aristotle, and Galen. Alliot kept the humoral theory, but he adapted it to the current discoveries. For example, the four humors became blood, lymph, chyle, and “nervous juice” [10]. He explained cancer as an unexpected mixture of two humors that creates a misbalance in the human body. He defined the cancer as follows:

*“cancer is a tumor, very hard, stony sometimes uneven and livid, always accompanied by pain more or less violent, depending on the circumstances that arise encounter, are more or less annoying.”*

According to him, there were two major causes of cancer:

- External (after a physical or emotional shock). He evoked the environmental influence (through polluted air or a contaminated food) that changes humor composition.
- Internal (misbalance between two humors). He evoked inheritance by describing some cases of similar symptoms between mothers and children [10].

In the second part of his treatise, he criticized the theory of Helvetius, a famous Dutch practitioner of that time who reduced the cancer origin to a single external shock, which appeared wrong for Alliot [10]. In a third chapter, Alliot proposed some recipes of solutions fighting against the “acid” to treat cancer. Additionally, for the first time, he proposed to carefully analyze the physical and moral state of the patient before deciding on the nature of the treatment [10] (Figure 3). To conclude, this important book had two functions: it summarized all the knowledge of antiquity to explain the origin of cancer, but it also crystallized all the criticisms of the new theories based on observations. Its edition paradoxically marked the end of the humoral theory (Figure 3).

## 3. From 1850 to 1885, Cancer Was Explained by the Theory of Spontaneous Generation

### 3.1. When the Circulation of Blood Puts an End to the Bloodletting Practice

While the Kingdom of France was consumed by the suffering of Queen Anne of Austria, the Kingdom of England took a major step forward in understanding the functioning of the human body with the physician William Harvey. Harvey belonged to the group of the privileged physicians of his time who were allowed to perform autopsies under the protection of their illustrious patients: he served as the physician of King James I and later King Charles I, as well as of the famous scholar Francis Bacon. During his studies in Italy, he met Girolamo Fabrizi d’Acquapendente who discovered the heart valves, which influenced his own research on the cardiac system. His numerous observations and avant-garde experiments helped him highlight the circulation of blood [11,12]. All these observations were gathered in his *De motu cordis* (“About heart movements”), published in 1628 [13]. However, the importance of this book for medicine emerged more slowly in continental Europe, since the concept of blood circulation challenged the prevalent practice of bloodletting, which formed the basis of medical treatments at that time. Indeed, because the bleeding points were located according to the diseased part of the body, bloodlettings no longer made sense as treatments if the same blood circulated throughout the body [11,12]. This new theory took longer to be adopted as it was not the first of its kind. Indeed, the doctor and theologian Michael Servetus, born Spanish and naturalized French, had already mentioned 50 years before Harvey, the possibility that blood circulates in the lungs, drawing inspiration from Arabic writings of the 13th century [14]. However, Michael Servetus also had very critical positions towards the Catholic Church and the dogma of the Holy Trinity. He was thus condemned to death and burned alive with all his books in Geneva, Switzerland in 1553 [14]. This is why few copies of his medical work have survived and Harvey’s work stood out as the precursor of a new theory. Moreover, this period was rich for the progress of knowledge in general because it was also during this time that the Newton’s theories were published, calling into question those of Aristotle, which had been untouchable since the Middle Ages. All this wind of discoveries, based on rigorous observations and interpretations, was supported and propagated by Voltaire himself throughout Europe [15]: the influence of the Catholic Church was then systematically contradicted or even defeated in a number of scientific and medical areas.

### 3.2. When a Theory Chases the Other: From Humoral Theory to Spontaneous Generation

One might have expected that, under the influence of Francis Bacon and René Descartes, the methodical and scientific works of Harvey could overcome the humoral theory. Even if this was the case, however, Harvey and Descartes were also the defenders of another theory developed by Aristotle, the theory of *spontaneous generation* [16]. This theory was based on the hypothesis that living beings find their origin without ancestry (*abiogenesis*). Hence, cancer may not be caused by a flow of acid on the flesh due to an excess of black bile as the humoral theory stated, but rather from germs *spontaneously* born from the depths of the body. For the Church, nothing really changed since the figure (and the ideas) of Aristotle remained an indisputable reference, even if a different theory was highlighted. With the theory of spontaneous generation, the notion of cancer as a divine punishment persisted. However, scientists like the Dutch biologist Jan Swammerdam, who was the first to introduce the use of the microscope, opposed the theory of spontaneous generation at the end of the 17th century [17,18,19]. In 1669, using a microscope, he described the metamorphosis of insects as well as the eggs of the queen bee, suggesting that each animal species obeys a life cycle from the egg to adulthood. Interestingly, the theory of spontaneous generation was more rarely cited for vegetal species and cereals while it was still widely believed until 1850 that maggots were born “from rotten meat” and that mice could descend from “a pile of old clothes”. Any farmer knew how to cultivate cereals, from the seeds to the plants, while the origin of animals could appear more obscure [17,18,19].

## 4. From 1895 to 1940, Cancer Was Explained by the Microbian Theory

### 4.1. The Death of the Spontaneous Generation Theory

**Figure 4:**
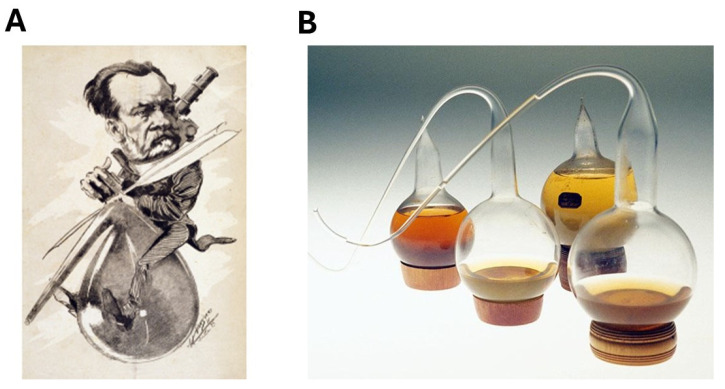
The theory of spontaneous generation and the microbian theory. (**A**) Caricature of Louis Pasteur by Luque in 1884, illustrating his works on spontaneous generation and fermentation. (**B**) A series of gooseneck balloons used by Pasteur to demonstrate the irrelevance of the theory of spontaneous generation (source: Institut Pasteur, Paris, France): a boiled broth did not provide any new life, while if air has some access to the solution, some microbes (germs) can grow.

Freethought appeared for the first time in 1850, in a speech by Victor Hugo, in which he advocated for independent thinking free from any dogma: it became easier to provide criticism to the religious dogmas. Furthermore, considerable advances were made in medical and biological sciences during the 19th century. Consequently, the theory of spontaneous generation became less and less defendable. Nine years after the speech of Victor Hugo, the relevance of spontaneous generation was as the center of a famous debate between Louis Pasteur and Félix Pouchet at the French Academy of Sciences. Through his rigorous experiments, Pasteur was one of the first scientists who demonstrated the existence of microbes: the theory of spontaneous generation was discredited definitively, and Pasteur’s germ theory, also called *microbian theory,* triumphed and became the reference [17,18,19] (Figure 4). Pasteur’s discoveries opened a new area in microbiology and parasitology. In the meantime, tuberculosis, which was a major cause of death in the whole population, was found to be of microbian origin in 1882, thanks to Robert Koch [20,21]. The fact that a number of other diseases were also shown to be caused by micro-organisms convinced a great majority of scientists and physicians that the microbian theory was universal. Consequently, through this enthusiastic movement of progress, cancer was also considered to be caused by a contamination due to micro-organisms. This was the microbian (germ) theory of cancer [22,23].

### 4.2. The First Anti-Cancer Radiotherapy: An Example of the Predominance of the Microbian Theory

As specified above, during the second part of the 19th century, nearly all the diseases, including cancer, were considered to be caused by microbian (germ) contamination. Interestingly, a historical event illustrated this erroneous predominance of the microbian theory: the first attempt of an anti-cancer radiotherapy by Victor Despeignes, only six months after the discovery of X-rays by Wilhelm Roentgen [24]. Despeignes, born in Lyon (France), in a familial environment convinced by the microbian theory of Pasteur, conducted brilliant studies at the University of Lyon. He later moved to Savoy, working as a simple general practitioner in a small village called the Echelles. In March 1896, his former colleagues from the University of Lyon applied X-rays to rodents, whose legs were injected with tuberculosis extracts. Only the irradiated rodents survived, suggesting for the first time that X-rays may have a curative action on microbes [24]. In early July 1896, at the Echelles village, a neighbor of Despeignes suffered from a tumor growing in his abdomen. Despeignes diagnosed stomach cancer and considered that this cancer, as with any disease at these times, was caused by micro-organisms according to the microbian theory of Pasteur. He therefore asked his former colleagues of Lyon to provide him with an X-ray tube to apply the same treatment as they did to rodent legs with tuberculosis, hypothesizing that the stomach cancer should be sensitive to X-rays, like tuberculosis [25,26,27]. For 22 days, Despeignes applied X-rays to the tumor twice a day. The tumor volume decreased significantly but the patient succumbed at the end of July 1896. In three articles, Despeignes described the reactions and the effect caused by X-rays on his patient [25,26,27]. In the first sentences of his first article, Despeignes emphasized that he began the radiotherapy *convinced by the microbian nature of the tumor*. However, a careful analysis of the description of Despeignes has revealed today that the cancer of his neighbor was likely to be a lymphoma due to his professional activities in the industry of viscose rayon, while the stomach cancer was later shown to be one of the rare cancer types caused by a microbe (*Helicobacter Pilori*).

### 4.3. The Cellular Theory, an Emerging Paradigm to Be Opposed to the Microbian Theory

**Figure 5:**
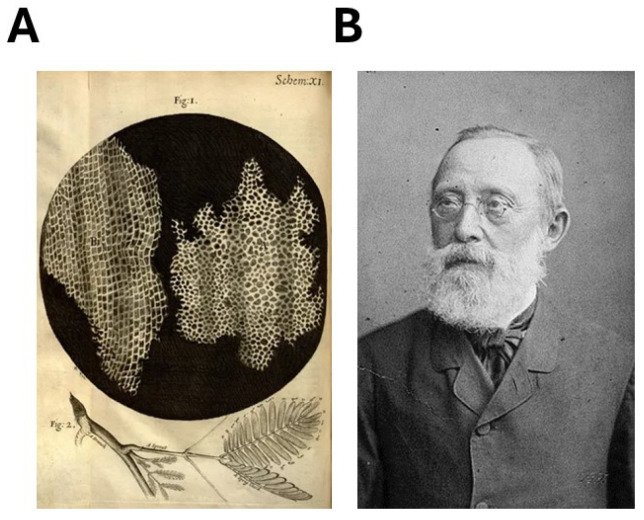
The cellular theory. (**A**) Robert Hooke’s drawings, from a microscopic view, of cork and a sprig of a plant, from *Micrographia* (1665). (**B**) Rudolf Ludwig Karl Virchow. Photograph by J. C. Schaarwächter, 1891.

In the meantime, a number of histologists used microscopes to observe the life cycle of any type of cells. Interestingly, the concept of the “cell” was born with the first microscopes and the microscopic observations of Robert Hooke, considered as the first scientist to describe vegetal cells in 1667. He chose the name “cell” as an analogy with the monks’ cells in monastery [28,29] (Figure 5). Eight years later, Antoni van Leeuwenh, a Dutch biologist, described living cells with an improved microscope [30]. Thereafter, a long series of microscopic observations consolidated the paradigm that cells are independent entities with a nucleus and a cytoplasm, whether vegetal or animal, and that they compose all living beings. The direct consequence of such observations was that “a given cell comes from another one by cellular division”. This was the conclusion of Rudolf Virchow in 1855 [31,32] (Figure 5). Wirchow was one of the students of Johannes Müller who probably made the most complete series of microscopic descriptions of tumor cells of that time. He notably identified benign and malignant tumors and concluded that they both are made of cells, like normal cells. However, probably still influenced by the microbian theory, he proposed that tumor cells originated not from microbes but from “tumor germ cells”, “pre-tumor cells”, or “tumor stem cells” that are scattered among normal tissue elements. Seven years after the publication of the works of Müller about tumors, Virchow augured a kind of compromise between the microbian (germ) theory (“cancer is due to contamination with micro-organism”) and the cellular theory (“cancer is due to deriving cell”): from 1855 to 1896, while the microbian (germ) theory of cancer was still opposed to the cellular theory of cancer *for the origin of the disease* (primum movens), Virchow stated that tumor cells, once “initiated”, obey the cellular theory of cancer for their division and proliferation. In other terms, the controversies between the two theories remained in the first divisions of the tumor but not in its evolution for which the microbian theory appeared irrelevant. It is also noteworthy that Virchow made a connection between inflammation and cancer [31,32].

## 5. From 1895 to 1940, Cancer Was Also Explained by the Cellular Theory

### 5.1. First Identifications of Carcinogenic Agents

In order to question the relevance of the hypothesis of a subset of “tumor germ cells” in the formation of tumor, considerable efforts were made to identify what physical, chemical, biochemical, or biological agent could induce cancer and impact the pool of tumor germ cells. From the second half of the 18th century, the observations of Sir Percival Pott, attributing cancers of the scrotum to the soot that stained the integuments of little chimney sweeps, demonstrated that an extrinsic agent could cause cancer [33]. At the end of the 19th century, the appearance of bladder cancers in dye factory workers was another example [34]. However, the most documented examples about carcinogenic agents were those involving X-rays. The first radiation-induced cancers were described in 1902 by Frieben and by pioneers of radiation: for the first time, a physical agent, alone, may induce cancer and its progression was explained by an uncontrolled cellular proliferation [35]. Furthermore, since physicists pointed out the stochastic nature of impacts on matter as radiation-induced energy microdepositions, it became clear that “tumor germ cells” could not be impacted specifically by radiation: cancer could be induced by X-rays without impacting on any pool of irradiated “tumor germ cells”. In other terms, the microbian theory was irrelevant for the formation of radiation-induced tumors. Such conclusions weakened the relevance of the tumor germ cells theory to the rare case of viral or microbian contamination and revealed the stochastic nature of cellular targeting when the carcinogenic agent is physical, chemical, or even biochemical [22,36].

### 5.2. When the Chromosome Appeared and When X-rays Changed the Situation Again

**Figure 6:**
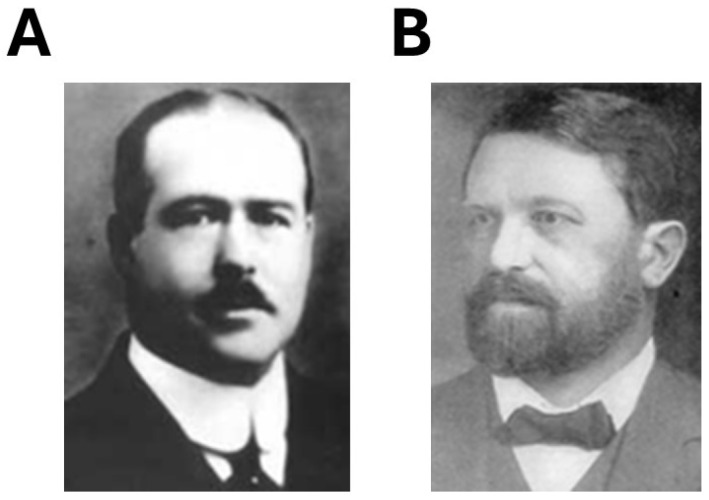
(**A**) Walter Sutton. (**B**) Theodor Boveri.

Theodor Boveri was one of the first biologists who stated that cancer originated from a single deriving cell. He attributed the lack of control of proliferation to one chromosome aberration. Boveri developed a new theory that became one of the most important of the 20th century: the chromosome theory, also called the Boveri–Sutton theory, since the American Walter Sutton converged to the same conclusion at the same time [37,38]. Boveri studied the chromosomes of sea urchins while Sutton chose grasshoppers as his study model. They both concluded on the existence of a genetic material composed of chromosomes, transmittable over generations and potentially sensitive to damaging environments (Figure 6). Interestingly, while Boveri worked at the same University as Roentgen, the University of Würsburg (Germany) and they were close friends, Boveri did not use X-rays to verify the relevance of his theory with a physical carcinogenic agent [39,40]. It would take several decades and the work of Claudius Regaud and Hermann Muller on irradiated corn to better understand the effects of ionizing radiation on carcinogenesis (see next chapters). The evolution of the ideas about carcinogenesis at the beginning of 20th century can be summarized as follows:

- Cancer is known to follow several ordered steps of evolution.
- Some cancer types may be caused by micro-organisms (like stomach cancer) but the majority of them appeared to be caused by one of several deriving cells.
- Some scientists hypothesized the existence of tumor germ cells that may derive preferentially. However, investigations with X-rays called into question the existence of a pool of germ cells since exposure to X-rays induces cancer but impacts cells indifferently.
- The chromosome theory suggested that, whether in the tumor germ cells or in any cell, the primum movens of the formation of tumor is explained by one chromosome aberration per cell.

## 6. From 1895 to Today, Cancer Is Also Explained by Chromosome Theory

### 6.1. The Tribondeau and Bergonié Law: A Nice Formula That Propagates a False Idea

At the beginning of the 20th century, in the histology lab of Pr Renaut, Claudius Regaud developed a staining solution that permitted the detection of mitochondria [41]. This solution, known as Regaud’s staining solution, was applied to the irradiated testicles of rats by Jean Alban Bergonié, the most famous French elecroradiologist of that time and Louis Tribondeau, a military practitioner [42]:

*“… in our experiments, on the testicles of the rat, we have been able to destroy the germinal cells whereas the interstitial tissue and the Sertoli syncytium remained intact. Thank to these results, it has been possible to formulate the following law: X-rays act on cells inasmuch efficiently as cells have a greater reproductive activity, their karyokinetic fate is longer, their morphology and function are least definitively fixed.”…”Hence, from this law it is easy to understand that roentgenisation destroys tumors without destroying healthy tissues…”*.

This law, and particularly the last sentence, became the scientific basis of radiotherapy. Surprised to see his own staining solution linked to statements that he thought to be wrong, Regaud was the first to provide counter examples against the Tribondeau–Bergonié law. He demonstrated that the generalization of this law to tumor cells was not correct in radiotherapy; in fact, cellular radiosensitivity is independent of the proliferation power of cells [43]. Unfortunately, even today, this erroneous formula is currently cited in lectures.

In the second part of the same paper that Tribondeau and Bergonié published in 1906, in a part often neglected by readers, the authors stated that, while a high dose of radiation can cure cancer, low and repeated doses may favor it [42]:

*“It seems pretty clear that the practice of delivering small and repeated doses, in contradiction to the technique with less frequent and heavy doses, is more apt to produce these nondestructive irritations resulting in giant cells and probably malignant transformation.”*

Interestingly, such an observation suggested that cell transformation and cancer not only compete with cellular death but may be favored by repeated sublethal doses. Again, such a statement does not require the existence of a pool of tumor germ cells but suggests that low and repeated doses of ionizing radiation may alter the cell nucleus and favors cellular proliferation [42,43].

### 6.2. The Works of Claudius Regaud and Hermann Joseph Muller with X-rays

Paradoxically, the works of Bergonié and Tribondeau on irradiated cells with his own staining solution encouraged Regaud to focus on radiobiology while he was interested in histology. He investigated the radiosensitivity of all the cells composing the male and female reproductive systems. In 1908, he established the first laws of radiobiology: he suggested that chromatin is the most radiosensitive part of the cell, X-rays can produce cell cycle arrest and confirmed that low doses of X-ray may induce some long-term cellular transformations. Hence, Regaud consolidated the first observations made by Tribondeau and Bergonié [44].

In addition, Regaud highlighted an analogy between the cell progeny in the male and female reproductive systems and the succession of cellular steps in cancer progression, suggesting a hierarchy and a progeny between the first cells irradiated and the last daughter cells of their descendance. His works suggest a long transmission of the chromosome features of the cells irradiated and their amplification in the daughter cells [44,45,46] (Figure 6).

However, until the end of his career, Regaud hesitated between the microbian theory and the cellular theory to explain cancer. In 1941, he wrote, seven months before he died, his last paper about the pathogeny of cancers and these two theories. Regaud was unable to decide between the two theories [47]:

*“I was tempted to publish this dissertation in a collection of oncology work: this would have had the advantage of making my ideas available to all researchers. I will not do it, because the fabric of my hypotheses is perhaps only a work of imagination; and, even if the future were to demonstrate that such a fear was unfounded, I will not do so because, having undertaken this work too late, I have not been and will no longer be able to give it the substance and form, which are necessary in a scientific publication. In particular, I was unable to gather the bibliographic documentation which would allow me to link my own ideas to the numerous works of my predecessors on the same subject…. Intermediate between publication and silence, the form of this writing seemed to me to correspond to the meaning of what it contains.”*

Another example of work with X-rays that significantly contributed to cancer research was conducted by Hermann Joseph Muller. In Thomas Morgan’s lab, Muller investigated the mutations in Drosophila, first treating them with heat and, then with X-rays. The experiments of Muller were quantitative and in 1926, he demonstrated that X-rays induce mutations by obeying a specific function of dose. He was awarded the Nobel Prize for his work on radio-mutagenesis, and a link was established between the occurrence of radiation-induced cancer, the yield of radiation-induced chromosome aberrations, and the dose of radiation [48].

## 7. From the 1940s to the 2000s, Initiation, Promotion, and Progression Became the Holy Trinity of Carcinogenesis

### 7.1. The Definition of the Multi-Step Formation of Cancer

Quantitative assessments of molecular damage were first demonstrated with cytogenetics. The discovery of the DNA structure in 1953 permitted, some decades after, to consolidate these conclusions with DNA sequencing. Hence, whether with cytogenetics or DNA sequencing, the data about mutagenesis encouraged scientists to develop new theories about carcinogenesis by focusing on the multi-step formation of cancer, i.e., *independently of the microbian or cellular nature of its origin*, since the timing of the processes involved became of crucial interest. In addition to this, the microbian and cellular theories of cancer were mentioned less and less in the literature. In 1941, Isaac Berenblum who studied the carcinogenesis caused by the dimethyl benzanthracene (DMBA) on the skin of rodent models proposed three independent phases for carcinogenesis [49]:

- A preneoplastic phase (or precarcinogenic action);
- A “wart stage” (or epicarcinogenic action);
- A malignant transformation of these warts (or metacarcinogenic action).

By studying the effects of the same DMBA on the skin of rabbit models, William F. Friedewald and Peyton Rous in 1944 proposed to change the terms introduced by Berenblum for the first two steps as “initiation” and “promotion”, respectively [50,51]. In one of his papers published in 1947, Berenblum agreed to this proposal [52]. For the third step, the term “progression” had been chosen some years before. Indeed, it is generally said that Rous and Beard in 1935 were the first to introduce it to describe the development of carcinomas from virus-induced papillomas in rabbits [53]. The notion of tumor progression was thereafter developed by Foulds who proposed six rules [54,55,56], revisited by Rubin in 1994 [57]:

- Rule I: Progression occurs independently in different tumors in the same animal.
- Rule II: Progression occurs independently in different characters in the same tumor.
- Rule III: Progression is independent of growth. It occurs in latent tumor cells and in tumors whose growth is arrested.
- Rule IV: Progression is continuous or discontinuous, by gradual change or by abrupt steps.
- Rule V: Progression follows one of alternative paths of development.
- Rule VI: Progression does not always reach an end point within the lifetime of the host.

The advantage of the triptych “initiation, promotion, progression” in carcinogenesis was the simplification of the description of this obscure multi-step evolution from premalignant cells to invasive cancer. Scientists were then able to focus on one step of this triptych and a considerable number of variant theories of carcinogenesis which are difficult to overview, emerged. A certain consensus around these three terms began to be established.

### 7.2. New Concepts and the Two-Hit Hypothesis

Since the 1960s, numerous biologists have conducted fusions of cancer and normal cells to investigate whether the “oncogenic” character was dominant or recessive. While they obtained various responses based on their protocols and the tumor types investigated, these biologists shared common terms. *Tumor suppressor genes* were firstly defined as genes whose mutations confer a *cancer predisposition* (a concept initiated by Harris in 1969 [58]). *Oncogenes* were firstly described as genes involved in cellular proliferation with one of the most representative examples being provided by Stehelin, Varmus, Bishop, and Vogt in 1976 with the *src* sequences of the Rous sarcoma virus [59]. Briefly, the proto-oncogenes normally activate cell growth and division and maintain homeostasis, while mutations of proto-oncogenes can activate oncogenes, leading to the lack of control of cellular proliferation and the development of cancer. The study of oncogenes culminated with the 1989 Nobel Prize in Physiology and Medicine attributed to Michael Bishop and Harold Varmus [60,61].

In response to the discrepancies found in the results of fusion between oncogenic and normal cells, Alfred G. Knudson pointed out that most genes whose mutations lead to cancer required the inactivation of both alleles. In 1971, with the retinoblastoma gene (Rb), Knudson noticed that inherited retinoblastoma occurs earlier than sporadic cases, suggesting that the inherited heterozygous *Rb* mutation requires a second “hit” to trigger the formation of cancer by inducing an homozygous mutation [62]. This two-hit hypothesis was also compatible with the transformation process of a proto-oncogene to an oncogene [62]. By contrast, haploinsufficiency could explain the occurrence of cancer from heterozygous mutations (e.g., *BRCA1*/*BRCA2* mutations and inherited breast cancers). The term “anti-oncogene” was introduced from 1986 with the discovery of the retinoblastoma gene (*Rb*) [63,64].

### 7.3. The Major Hypotheses of the Initiation Step

From the 1970s, two hypotheses (or submodels) were cited to characterize the initiation step [65]:

- The stochastic submodel based on the hypothesis that each cell is potentially able to form a tumorigenic clone. The hypothesis that the primum movens, whether due to a micro-organism or occurring in a cell, was one of several DNA/chromosome mutation(s) (according to the chromosome theory) and was at the center of this submodel. Some endogenous factors (like genomic instability and individual predisposition) may be involved to lead to a subset of cells that are able to be amplified and form the “wart” stage.
- The tumor stem cells (or hierarchy) submodel was based on the hypothesis that only a specific subset of cells is able to reach the “wart” stage. This submodel summarized the previous tumor germ cells or precancerous cells models cited above. The notion of tumor stem cells was notably refined and born from the studies about the reproductive or hematopoietic systems in which a hierarchy in the cellular differentiation was observed (e.g., Regaud’s works) [65]. The tumor stem cells should be auto reproductive to ensure, at any time, a certain pool of cells (reservoir). However, the notion of tumor stem cells was not consensual. A number of specific biomarkers of tumor stem cells (notably some biomarkers linked to the cellular surface state) were identified and thereafter abandoned [66,67,68]. Furthermore, as mentioned above, ionizing radiation does not impact the tumor stem cells pool preferentially while they induce cancer, which raises questions about the existence of tumor stem cells. In addition to this, a viral or bacterian infection raised similar questions, suggesting that the microbian theory was not necessarily compatible with the tumor stem cells submodel.

During this period, the number of DNA/chromosome mutations required per cell to enable the promotion step became an important subject of investigations. It was generally considered that a mutation (or an epigenetic event) can spontaneously occur in one cell every three cellular divisions [38,69]. Such incidence is very low: exogenous or endogenous factors are therefore needed to amplify this number (e.g., Muller’s work). The very first mutation (the *primum movens*) may be due to misrepaired DNA breaks caused by a significant amount of oxidative stress induced by coincidence, environment (radiation, chemicals, virus, …) or by a certain predisposition to misrepair DSB (DNA mutations, founding effect). However, unlike the hypothesis of Boveri, it appears clear that one mutation per cell cannot produce a tumorigenic clone: several other mutations are required [38]. At this stage, it is unlikely that this second series of mutations would be created randomly. With regard to the exogenous factors, there is evidence that repeated exposures of low stress can produce several mutations. With regard to endogenous factors, a familial predisposition may ensure a sufficient mutations pressure to produce several mutations per cell. However, the mixed contribution of both types of factors is also possible (e.g., some mutations induced by exogenous factors followed by some mutations by endogenous factors, or reciprocally). During the 1940–1970 period, some scientists like C.O. Nordling calculated the minimal number of mutations per cell required to initiate cancer [69]. They notably based their calculation by considering the age at which cancer occurs. They concluded with the following [69]:

- One mutation per cell is not sufficient to initiate cancer;
- Three to seven mutations per cell is the minimum number of mutations required to initiate cancer;
- Cell proliferation is needed to amplify the number of mutations per cell;
- The frequency of carcinoma increases according to the sixth exponent of age.

### 7.4. Caretakers, Gatekeepers, and Landscapers

Between the 1960s and the 1990s, the term “tumor suppressor genes” was essentially used for describing the genes involved in the control of cellular proliferation. However, in order to integrate the genes that were likely involved in DNA damage repair and signaling and whose mutations led to high cancer risk, Kinzler and Vogelstein proposed in 1997 that tumor suppressor genes could be divided into two major categories, known as caretakers and gatekeepers [70].

- Caretaker genes are involved in the maintenance of genome integrity and stability and include genes implicated in DNA repair: when mutated, caretaker genes explain the accumulation of mutations in cells.
- Gatekeeper genes directly regulate cell growth by either stimulating or inhibiting proliferation, differentiation, or apoptosis.

Interestingly, this classification allowed for correspondences to be established with the triptych “initiation, promotion, progression”, with caretakers aligned with initiation and gatekeepers with promotion. However, this view remains simplistic since gatekeeper suppressor genes might contribute to the dysregulated signaling pathways, as well. In 2004, Franziska Michor, Yoh Iwasa, and Matin Nowak introduced the notion of landscaper genes that, when mutated, may contribute to the environment of the tumor by favoring its growing [71,72]. Hence, at the beginning of the 2000s, a long succession of ideas, notions, and technologies had emerged, forming a double triptych that may appear imperfect but gathers the major principles of carcinogenesis.

## 8. Since the 2000s, Refinement of an Existing Model or New Theory?

### 8.1. Addition of Cell Hallmarks to Current Models

Since 2000, Hanahan and Weinberg’s work in defining the hallmarks of cancer has been foundational in understanding the multi-step process of carcinogenesis required to reach a clinically detectable tumor [2,3,73]. Initially, they identified six hallmarks required for tumor development: limitless replicative potential, evading apoptosis, self-sufficiency in growth signals, insensitivity in anti-growth signals, sustained angiogenesis, and tissue invasion and metastasis. This biophysical model also does not specify by what mechanism these characteristics are acquired or in what order they appear, but they all seem necessary. These hallmarks represented key biological capabilities acquired, mostly during tumor progression. In 2011, Hanahan and Weinberg revised their original definition of the six original hallmarks (sustaining proliferative signaling; evading growth suppressors; resisting cell death; enabling replicative immortality; inducing angiogenesis; activating invasion and metastasis) and added two more hallmarks: reprogramming of energy metabolism and evading immune destruction [3]. They also recognized that the acquisition of the hallmarks requires the creation of a favorable tumor micro-environment at the tissue level by normal cells. In 2022, Hanahan again introduced potential refinements in the comprehension of cancer mechanisms [73]:

*“Hence, the prospect is raised that phenotypic plasticity and disrupted differentiation is a discrete hallmark capability, and that nonmutational epigenetic reprogramming and polymorphic microbiomes both constitute distinctive enabling characteristics that facilitate the acquisition of hallmark capabilities. Additionally, senescent cells, of varying origins, may be added to the roster of functionally important cell types in the tumor microenvironment.”*

Although the hallmarks model is not mechanistic, it seems possible to make attempts of correspondence with initiation, promotion, progression on the one hand, and caretakers, gatekeepers, and landscapers on the other hand.

### 8.2. Specific Complements for the Progression Step

In the same efforts to refine the existing models, the progression step may have been enriched by three specific features, required for the amplification of the tumor volume at the tissue scale:

- Tumor microenvironment (TME): In a tissue, cells communicate together and with the surrounding stroma, either directly, through junctional complexes, or indirectly through factors secreted in the environment extracellular (hormones, factors of growth, chemokines, etc.). Some of these factors may favor cancer progression. Notably, tissues in proximity of some invasive cancers (e.g., adipose tissue), may contribute to cancer development through their capability to secrete pro-inflammatory cytokines [74,75].
- Tumor blood supply: Cavallaro and Christofori (2000) addressed the important role of angiogenesis in the growth of solid primary tumors as well as their metastases. Induction of angiogenesis precedes malignant tumor formation, and increased vascularization appears to be associated with the invasive properties of tumors, thus defining the malignant tumor phenotype [76]. Although the discovery of tumor-derived angiogenesis modulators has greatly improved our understanding of tumor regulation of angiogenesis, the connection between angiogenesis and tumor progression remains misunderstood. In parallel to angiogenesis, blood supply could be addressed by the de novo vessels channel developed from cancer cells transdifferentiated into endothelial cells [77].
- Epithelial–mesenchymal transition (EMT): EMT is an embryonic capability through which epithelial cells lose many characteristics insuring homeostasis and acquire new characteristics of mesenchymal cells. EMT mechanisms may be reactivated during cancer progression, allowing tumor cells to enhance their motility and invasiveness. In vitro and in vivo observations suggest that EMT could favor both tumor development and metastatic dissemination [78,79].

### 8.3. The Bad Luck Hypothesis

More recently, a high-throughput approach has permitted evaluation of the variation in cancer incidence across different tissues and has revealed the following important statements [80,81,82]:

- The lifetime risk of patients being diagnosed with specific cancers can be very different according to the nature of the tissues and according to factors of exposure to certain physical (e.g., UV, radiation), chemical (e.g., drugs, smoking, alcohol) or biological (e.g., virus) agents. For example, lifetime risk is 6.9% for lung cancer in smokers. It is drastically reduced for non-smokers.
- The genetic factor enhances the risk of cancer occurrence but only 5 to 15% of cancers are considered to have an inheritable component.

However, the environmental and genetic factors remain insufficient to explain all the cancers, which have suggested to some authors the “bad luck” hypothesis, that would be due to random mutations arising during DNA replication in normal, noncancerous stem cells. Indeed, Tomasetti et al. have shown in 2015 that the relationship between the number of normal stem cell divisions and the risk of cancer was strongly correlated regardless of their environment [83]. The major role of the random (R) mutations was supported by cancer genome sequencing analysis and epidemiological data: the R mutations were found to be responsible for two-thirds of the mutations in human cancers. Although further investigations are needed to consolidate this conclusion, these findings may significantly impact on national public health policies [81].

### 8.4. The Epigenetic Investigations

While the above sections have shown the importance of DNA mutations in cancer formation, epigenetic alterations, i.e., chromatin-based events that regulate DNA-templated processes, may also influence carcinogenesis [84,85]. A total of 4 different DNA modifications (5-methylcytosine, 5-hydroxymethylcytosine, 5-formylcytosine, and 5-carboxylcytosine) and 16 classes of histone modifications have been identified (notably methylations, phosphorylations or acetylations) [84,85]. Such modifications may amplify and accelerate the DNA mutagenesis process and increase genomic instability. However, the specific endpoints that would be related to cancer formation are still to be defined. For example, is the total hyper-methylation of the genome of certain genes correlated to cancer proneness? To date, it appears clear that carcinogenesis is likely to involve genetic alterations, even if the hallmarks of cancers cited above involve the epigenome. However, it may be too early to define specific epigenetic endpoints as being included in mechanistic and mathematical models explaining the carcinogenesis process [84]. Further investigations are therefore needed to better understand the implication of epigenome in the occurrence of cancer [84,85].

## 9. Another Vision of the Carcinogenesis Process via the Interplay between Mutations and Proliferation

### 9.1. Cancer as a Protein Biokinetics Dysfunction

Normal cell functioning requires that each protein is the right (functional) protein, present in the proper amount, in the right place, at the right time. The antic hypothesis of misbalance to describe cancer can be therefore translated today as an alteration of the biokinetics of proteins that normally ensure genome integrity and control of the cell cycle. This paradigm may constitute a new vision of carcinogenesis by encompassing all the models described previously. Such a vision is notably supported by studies carried out in our laboratory since 2014 on the biokinetics of ATM, a major kinase protein of DNA damage repair and signaling, also required for genome maintenance, the control of the cell cycle, and the choice of cell death pathways [86]. In fact, in response to irradiation or any oxidative genotoxic stress, cytoplasmic ATM dimers monomerize and diffuse to the nucleus. Once inside the nucleus, they can phosphorylate specific substrates like H2AX histones on the DNA double-strand breaks sites, which triggers their recognition and repair. This model is named RIANS for radiation-induced ATM nucleoshuttling, or SIANS for stress-induced ATM nucleoshuttling [86,87]. Interestingly, the ATM monomers may be sequestrated in cytoplasm by some abnormally expressed ATM substrates called X-proteins and delay or impair the DNA damage recognition and repair function. If the nucleoshuttling of the ATM protein is not performed in due time (some minutes), toxicity, cancer or accelerated aging is observed [86,87]. The RIANS has been documented abundantly and provides, to date, one of the best predictions of post-radiotherapy radiosensitivity [88]. More recently, the RIANS/SIANS models have contributed to provide a new mechanistic explanation of degenerative diseases, most notably for Alzheimer’s disease [89].

### 9.2. The RIANS/SIANS Model and Carcinogenesis

**Figure 7:**
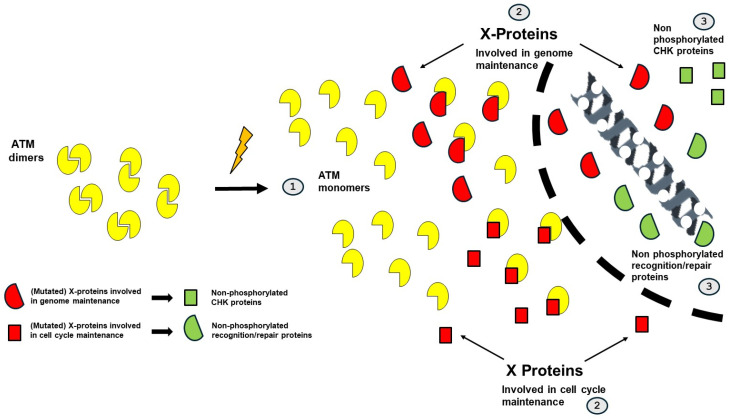
Schematic representation of the RIANS model to explain carcinogenesis. After exposure to radiation in particular and to genotoxic stress in general, ATM dimers monomerize (step 1). X-proteins delay the ATM diffusion in the nucleus (step 2). If the mutated gene is involved in the DNA recognition and repair proteins, DNA mutations are generated. The lack of ATM monomers in the nucleus does not allow the phosphorylation of the CHK proteins, which prevents the cell cycle checkpoints and favor cellular proliferation (step 3). If the mutated gene is involved in the cell cycle maintenance, cellular proliferation is favored. The lack of ATM monomers in the nucleus does not allow the activation of the DNA recognition and repair proteins, which favors DNA mutations (step 3).

In a recent review, we pointed out that the great majority of cancer syndromes are caused by mutations of genes that encode phosphorylation substrates of ATM, i.e., potential X-proteins. These proteins are either involved in DNA damage recognition and repair or in the control of cell cycle. The ATM kinase therefore appears at the crossroads of the molecular and cellular bases of cancer proneness [90]. As mentioned in this review, the following can be considered (Figure 7):

- The impairment of the ATM-dependent DNA damage recognition, signaling, and repair may cause misrepaired DNA damage that may be either endogenous (e.g., spontaneous DNA breaks) or exogenous (e.g., from the environment). Their formation can be considered as an initiation step in carcinogenesis.
- The impairment of the ATM-dependent cell cycle checkpoint control: impaired G2/M and/or G1 arrests may contribute to the propagation of the errors described above. This step can be considered as a promotion step in carcinogenesis.

Hence, in the frame of the RIANS/SIANS model, a given X-protein ensures its own intrinsic function as simple protein but plays additional biological role(s) as a cytoplasmic ATM substrate. Three categories of cancer syndromes have been defined as follows [90] (Figure 7):

- Category 1: Proteins whose mutations cause category 1 cancer syndromes are crucial for the integrity of the genome. In cells derived from these syndromes, the genomic instability is so high that numerous mutations are produced. In parallel, the cytoplasmic forms of the mutated protein, as X-protein, sequestrate ATM monomers in cytoplasm, which limits the phosphorylation of CHK1 and CHK2 proteins and inhibits the G2 and G1 arrests, respectively. Cellular proliferation is favored. A representative example of category 1 syndrome is Nijmegen’s syndrome, caused by *NBS1* mutations.
- Category 2: Proteins whose mutations cause category 2 cancer syndromes are crucial for the control of the cell cycle. In cells derived from these syndromes, cellular proliferation is favored. In parallel, the cytoplasmic forms of the mutated protein, as X-protein, sequestrates ATM monomers in cytoplasm, which limits the DNA damage recognition and repair and favors genomic instability and numerous mutations. A representative example of category 2 syndrome is Li-Fraumeni syndrome, caused by *p53* mutations.
- Category 3: Proteins whose mutations cause category 3 cancer syndromes are not necessarily crucial for both integrity of the genome and control of the cell cycle but, when combined with the role of the mutated proteins as X-proteins in cytoplasm, the resulting delay of ATM nucleoshuttling concerns both the integrity of the genome and the control of the cell cycle and favors cellular transformation. A representative example of category 3 syndrome is neurofibromatosis type 1 syndrome, caused by *NF1* mutations.

## 10. Conclusions

How does a normal cell transform into cancer? To address this fundamental question, a variety of theories of carcinogenesis have flourished through history. Progressively, technological and scientific advancements led to a final comprehensive mechanistic model in which three major steps have been identified: the initiation step, during which some impaired caretakers contribute to misrepair DNA damage and to produce DNA mutations; the promotion step, characterized by impaired gatekeepers that promote cellular proliferation, thereby amplifying the number of DNA mutations; and the progression step, where impaired landscapers contribute to cellular growth at the tissue scale. However, since the 2000s, while this final model appears relatively simple, several cancer hallmarks that are difficult to integrate have been pointed out. Hence, to date, the multiplication of hallmarks has paradoxically failed to consolidate our vision of carcinogenesis, inasmuch as the significance and prevalence of each hallmark remain elucidated incompletely. Considerable efforts are thus needed to further document the carcinogenesis process and attain a global and comprehensive understanding.
